# Research progress on regulatory mechanisms of mucosal barriers and their applications in allergic diseases

**DOI:** 10.3389/fimmu.2026.1671677

**Published:** 2026-01-27

**Authors:** Yipeng Zhang, Sheng Tian, Renzhong Wang, Yunhong Ning

**Affiliations:** 1 The First Clinical Medical College, Shandong University of Traditional Chinese Medicine, Jinan, Shandong, China; 2 Shandong University of Traditional Chinese Medicine, Jinan, Shandong, China; 3 Department of Otorhinolaryngology, Affiliated Hospital of Shandong University of Traditional Chinese Medicine, Jinan, Shandong, China

**Keywords:** allergy, JAK-STAT, MAPK, mucosal barrier, RhoA/ROCK, TGF-β/Smad, Wnt/β-catenin

## Abstract

The mucosal barrier, as a critical interface of the body’s defense system, is central to the pathogenesis of allergic diseases, with its structural integrity (epithelial cells, tight junctions, mucus layer, basement membrane) and functional homeostasis being key factors. This paper systematically elucidates the dynamic regulatory network constituted by five major signaling pathways: Wnt/β-catenin, TGF-β/Smad, RhoA/ROCK, MAPK, and JAK-STAT. These pathways interact through cross-talk (for example, Smad7 inhibits TGF-βRI to enhance Wnt signaling, and the β-catenin/Smad4 complex synergistically activates EMT genes), forming synergistic/antagonistic effects that jointly regulate epithelial repair, the expression of tight junction proteins (ZO-1/Claudin/Occludin), mucus secretion (MUC2/MUC5AC), and basement membrane remodeling. In allergic diseases, this network exhibits organ-specific imbalances: respiratory barrier damage is primarily characterized by RhoA/ROCK-mediated abnormal mucus secretion (asthma) and JAK-STAT-driven Th2 inflammation (rhinitis), whereas the intestinal barrier relies more on the epithelial regenerative capacity of the Wnt pathway.We innovatively propose a “phased-organ-targeting strategy”: during the acute inflammatory phase (0-72 hours), JAK inhibitors (such as CYT387 nasal spray) are utilized to block STAT6 phosphorylation and control the immune storm; in the repair phase (72 hours to 2 weeks), Wnt agonists (WNT2b-pH microspheres) are employed to promote epithelial regeneration, or RhoA regulators (fasudil inhalation) are used to reconstruct the mucus layer; in the chronic remodeling phase, a temporally regulated dual-pathway therapy (such as JAK-STAT inhibition combined with Wnt activation hydrogels) is applied. The current challenges lie in overcoming pathway redundancy, tissue delivery efficiency, and individual differences in microbial flora. Future efforts should focus on achieving precise interventions through local delivery using nanocarriers, temporally coordinated dosing regimens, and predictive models of microbiota-host interactions.

## 1 Introduction

The structure of the mucosal barrier is complex and intricate, primarily consisting of epithelial cell layers, tight junctions (TJ), mucus layers, and the basement membrane (1, 2). Its core functions are to defend against pathogenic invasion and regulate immune homeostasis. The barrier functions at various sites are precisely regulated by a series of signaling pathways, ensuring effectiveness by modulating epithelial cell proliferation, differentiation, barrier integrity, and immune responses. Different regions of the mucosal barrier face distinct environmental challenges, and their respective barrier functions are finely tuned through specific immune responses and signaling transduction mechanisms, thereby providing protection to the body and ensuring physiological functions. This article focuses on the regulatory mechanisms of key signaling pathways in the intestinal and respiratory mucosal barriers, with particular emphasis on their translational application value in allergic diseases.

## 2 Structure of the mucosal barrier

### 2.1 Epithelial cell layer with TJ

The epithelial cell layer is central to the mucosal barrier, primarily composed of one or more layers of epithelial cells that are interconnected by TJs, forming an almost impermeable barrier. TJs regulate the permeability between cells, preventing external substances, particularly pathogens and toxins, from crossing (3). In the intestine, tight junction proteins such as Claudin, Occludin, and JAMs play a crucial role. Literature (4) indicates that the expression of Claudin-1 and Claudin-7 is significantly reduced in patients with inflammatory bowel disease (IBD)(such as ulcerative colitis), leading to increased permeability of the epithelial barrier. This dysfunction of TJs directly results in the disruption of the mucosal barrier, thereby contributing to the persistence and exacerbation of inflammation.

### 2.2 Mucus layer

The mucus layer covers the surface of epithelial cells and is composed of mucins (such as MUC2, MUC5AC, etc.) and antimicrobial peptides. It not only protects epithelial cells from mechanical damage but also prevents infections by capturing and clearing pathogens (5). The mucus layer in the intestine is particularly important as it provides a living environment for the symbiotic bacteria while limiting the direct contact of pathogens with epithelial cells.

### 2.3 Basement membrane

The basement membrane is a thin membrane situated between epithelial cells and connective tissue, primarily composed of a translucent layer and a dense layer, containing components such as laminin and type IV collagen. It not only supports the adhesion of epithelial cells through its reticular structure, maintaining cellular polarity and providing mechanical support, but also serves as a selective barrier. Additionally, the basement membrane regulates cell proliferation and migration, promoting repair during mucosal injury by releasing growth factors, and modulating cell migration and differentiation through the activation of integrin signaling pathways (6). During injury or inflammation, the basement membrane undergoes remodeling via the action of matrix metalloproteinases (MMPs), assisting in the repair of damaged tissue and restoring barrier function. The dynamic changes of the basement membrane are crucial for mucosal regeneration and repair (7).

## 3 Physiological and pathological functions of mucosal barrier

The mucosal barrier is not only a mechanical barrier but also plays a crucial role in immune defense, nutrient absorption, and the maintenance of homeostasis through a series of complex physiological mechanisms. It serves multiple functions, including physical, immune, and chemical barriers. Dysfunction of the mucosal barrier is closely associated with the occurrence of various diseases, manifested by increased barrier permeability, inflammation, immune dysregulation, and allergic reactions.

### 3.1 Physical barrier function

The physical barrier function of the mucosal barrier primarily relies on the synergistic action of the epithelial cell layer and TJs. TJs regulate the permeability of intercellular spaces, preventing the passage of pathogens and toxins. The mucus layer enhances the physical protective role of the barrier by capturing and expelling pathogens (7, 8). When TJs are compromised or epithelial cells are ruptured, the permeability of the mucosal barrier increases, allowing pathogens, toxins, and inflammatory factors to penetrate the mucosa and enter the body. This situation is particularly common in various intestinal diseases, including IBD and functional bowel disorders (1, 2). These diseases are often characterized by significant changes in the physical and chemical properties of the mucosal barrier, leading to dysbiosis of the intestinal microbiota and chronic inflammation.

### 3.2 Immune and chemical barrier function

The mucosal barrier contains a rich array of immune cells, including macrophages, dendritic cells, T cells, and B cells. These immune cells are capable of recognizing and eliminating pathogens that enter the mucosa, playing a crucial role in immune and chemical defense. The mucus layer contains immunoglobulin A(IgA), antimicrobial peptides(AMP), and other immune-active substances, which neutralize or kill pathogens, preventing them from adhering to the surface of epithelial cells, thereby further enhancing immune defense and protecting the integrity of the mucosal barrier (8, 9). Studies have shown that the oral mucosa effectively prevents pathogen invasion through the continuous secretion of IgA (6). When the mucosal barrier is compromised, local immune responses may become excessively activated, leading to chronic inflammation or allergic diseases. For instance, in Allergic Rhinitis(AR) and Chronic Obstructive Pulmonary Disease(COPD), the function of the respiratory mucosal barrier is impaired, resulting in persistent infiltration of inflammatory cells and the release of a large number of inflammatory factors, causing tissue damage (10–12). Under normal circumstances, the mucosal barrier effectively prevents the invasion of allergens such as pollen, dust, and food proteins. However, when the mucosal barrier is damaged and permeability increases, these foreign substances can more easily penetrate the epithelial barrier, come into contact with the immune system, and trigger abnormal immune responses (10). Taking the respiratory tract as an example, damage to the mucosal barrier increases exposure to inhaled allergens, leading to the development of respiratory diseases such as AR and asthma (10, 13). Studies have shown that dysregulation of mucosal barrier function may lead to systemic chronic inflammatory responses, and maintaining the integrity of the mucosal barrier is crucial for the prevention and treatment of systemic diseases (10, 13).

### 3.3 Other functions

The mucosal barrier serves functions such as nutrient absorption, material exchange, and microbial symbiosis, with its selective permeability allowing the passage of nutrients, water, and gases to maintain ecological balance. For instance, the intestinal mucosa is capable of absorbing digested nutrients, while the respiratory mucosa facilitates gas exchange. Furthermore, the intestinal mucosal barrier closely collaborates with the symbiotic microbial community, which regulates the mucosal immune response through metabolic products (such as short-chain fatty acids) to prevent the colonization of pathogenic microorganisms. This balance between microorganisms and the host is crucial for maintaining the health of the mucosal barrier (5).

## 4 Regulation and influence of related pathways on mucosal barrier

The mucosal barrier can be divided into four interrelated structural components, namely the epithelial cell layer, TJs, the mucus layer and the basement membrane/extracellular matrix(ECM) (14–16). In this section, we focus on five representative signaling pathways that are closely involved in the regulation of these four layers: Wnt/β-catenin, TGF-β/Smad, RhoA/ROCK, MAPK(ERK, JNK, p38) and JAK-STAT. To provide a rapid global view and facilitate cross-comparison, **Table 1** summarizes these pathways in a matrix format.

**Table 1:** Overview of the regulatory patterns of key signaling pathways on the four-layer mucosal barrier.

Signaling pathways	Epithelial cells	Tight junctions	Mucus layer	Basement membrane/ECM
Wnt/β-catenin	↑ renewal/differentiation	↑↓ junction sealing (context-dependent)	↑ goblet cells & mucins	↑/↺ ECM synthesis & remodeling
TGF-β/Smad	↑↓ repair vs EMT	↑ homeostatic TJs; ↓ in chronic fibrosis	↑↓ via epithelial differentiation & inflammation	↑/↺ ECM deposition & fibrosis
RhoA/ROCK	↑ migration & restitution	↑↓ via cytoskeletal tension	↑ mucus secretion; imbalance if overactivated	↺ ECM remodeling (MMP-driven)
MAPK	ERK: ↑ proliferation; JNK/p38: ↑↓ stress response (survival vs apoptosis)	↑ TJ assembly (adaptive); ↓ when overactivated	↑ MUC expression; ↑↓ defense vs hypersecretion	↺ MMP/ECM balance; maladaptive remodeling if dysregulated
JAK-STAT	↑ survival, proliferation & antimicrobial peptides	↑ TJ proteins; ↓ with excessive Th2-biased activation	↑ MUC2/MUC5AC; ↑↓ mucus hypersecretion in chronic inflammation	↑/↺ regulation of MMPs & ECM; ↓ barrier strength if STAT–MMP/TIMP balance is disturbed

### 4.1 Effects on epithelial cells

**Figure 1:**
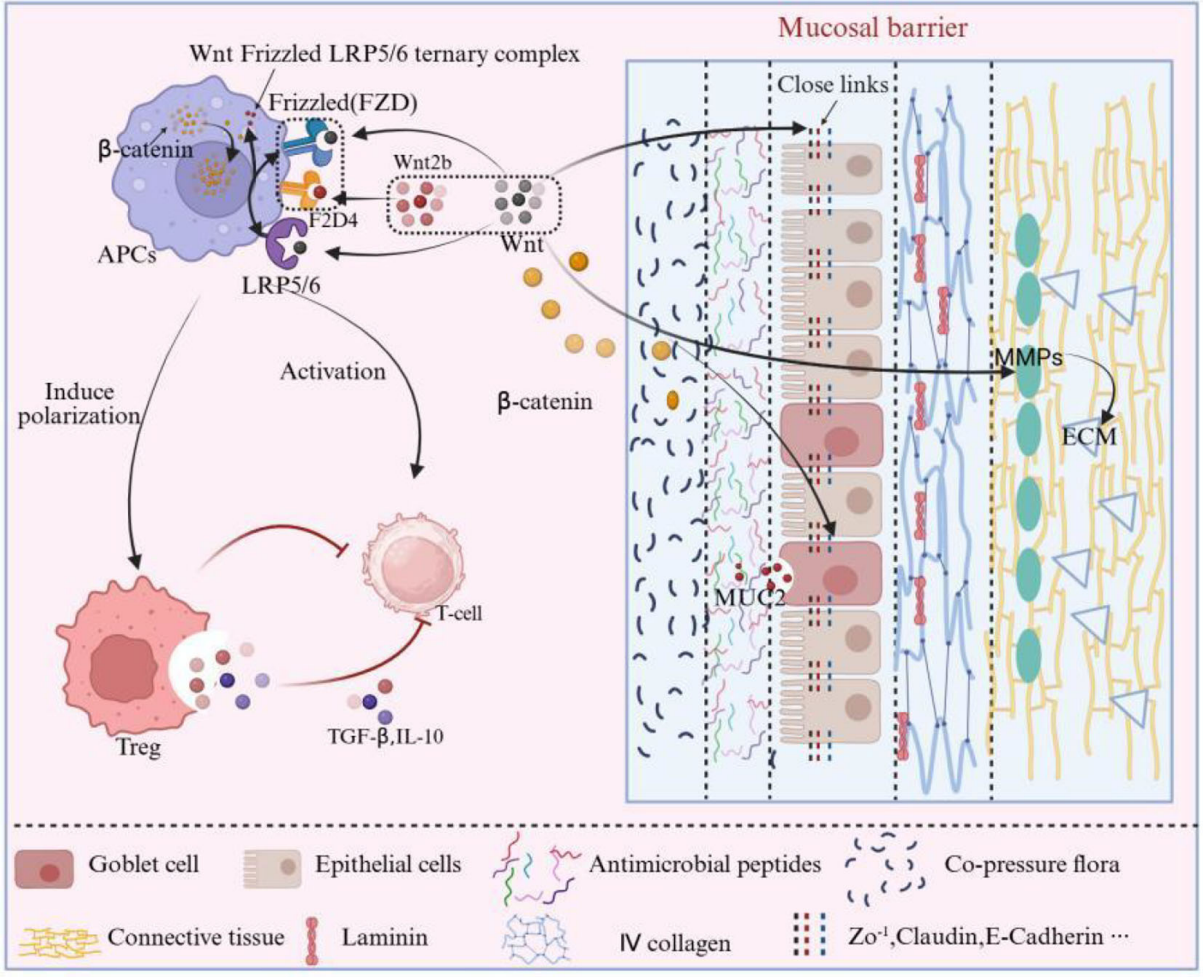
Wnt/β-catenin signaling pathway and its regulation of mucosal barrier components. The canonical Wnt/β-catenin pathway in epithelial cells: WNT2b and related ligands bind to the Frizzled–LRP5/6 receptor complex (for example FZD4–LRP5/6), inhibit the β-catenin destruction complex, promote cytoplasmic accumulation and nuclear translocation of β-catenin, and regulate target genes involved in epithelial renewal, TJ proteins, mucins, and extracellular-matrix remodeling. The figure also marks crosstalk with TGF-β/Smad signaling through Smad7 and the β-catenin/Smad4 complex, converging on EMT-related transcription factors such as Snail to fine-tune barrier repair. Key regulatory points (1) Epithelial cells: Wnt/β-catenin maintains epithelial stem-cell proliferation and differentiation, supporting continuous regeneration and homeostasis. (2) TJs: Wnt signaling context-dependently modulates the expression and localization of ZO-1, Claudins, Occludin and E-cadherin; in some tissues, high WNT2b–FZD4 activation is associated with reduced ZO-1/E-cadherin and weakened junctions. (3) Mucus layer: Wnt/β-catenin promotes goblet-cell differentiation and regulates mucin genes such as MUC2 and MUC5AC, contributing to mucus-layer formation and functional maintenance. (4) Basement membrane/ECM: Wnt signaling regulates MMPs and ECM components (for example fibronectin and type IV collagen) to participate in basement-membrane repair; its interaction with TGF-β/Smad (Smad7 and the nuclear β-catenin/Smad4–Snail axis) coordinates EMT intensity and barrier-repair efficiency.

The Wnt/β-catenin signaling pathway (mechanism detailed in **Figure 1**) maintains the homeostasis of the mucosal barrier by regulating the proliferation and differentiation of epithelial cells (17, 18). Studies have shown that the Wnt/β-catenin signaling pathway promotes the differentiation of Regulatory T Cells (Treg) by modulating the anti-inflammatory phenotype of Antigen-Presenting Cells (APCs), while simultaneously inhibiting the differentiation of Pathogenic Effector T Cells (pTeff), thereby maintaining intestinal immune homeostasis and supporting the functionality of the intestinal mucosal barrier (19). Research indicates that when Wnt/β-catenin signaling is inhibited, the proliferation of epithelial cells is halted, leading to the depletion of intestinal stem cells. The loss of these stem cells directly results in the depletion of intestinal epithelium and the disruption of its integrity (20).

Symbol legend: ‘→’ indicates activation/promotion; ‘┤’ indicates inhibition/negative feedback; ‘↔’ indicates synergy; ‘
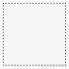
‘ indicates subordination.

**Figure 2:**
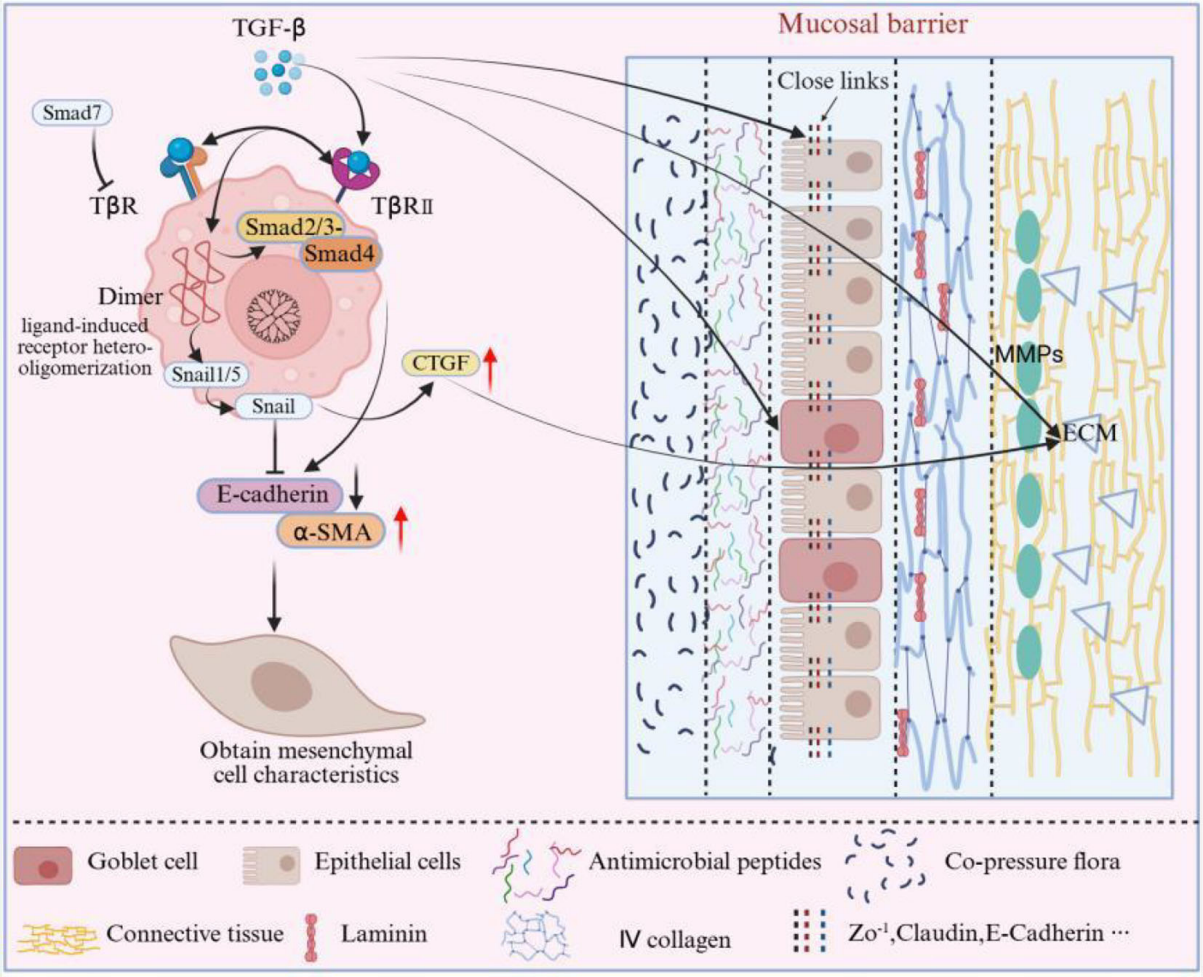
TGF-β/Smad signaling pathway and its regulation of mucosal barrier components. The TGF-β ligands bind to TβRII/TβRI on the epithelial membrane, forming an activated receptor complex (represented as a “Dimer”) that phosphorylates downstream Smads. The figure highlights two major branches: the canonical Smad2/3–Smad4 complex, which enters the nucleus to induce barrier-protective genes, and the Smad1/5–Snail branch, which drives EMT and fibrosis. Smad7 is shown as a typical negative-feedback inhibitor acting at the receptor level. Key regulatory points (1) Epithelial cells: TGF-β/Smad balances epithelial cell differentiation, apoptosis and EMT, exerting context-dependent protective or injurious effects on the barrier. (2) TJs: Under homeostatic conditions, the Smad2/3–Smad4 branch supports the expression of ZO-1, Claudins and Occludin; in fibrotic states, the Smad1/5–Snail–CTGF axis promotes EMT and collagen deposition, leading to tight-junction loss. (3) Mucus layer: By regulating epithelial differentiation and inflammatory mediators, TGF-β/Smad indirectly affects goblet-cell function and the expression of MUC5AC/MUC2, thereby influencing mucus-layer stability. (4) Basement membrane/ECM: TGF-β/Smad modulates ECM synthesis and remodeling via CTGF and MMPs (such as MMP-9); moderate activation favors repair, whereas persistent overactivation leads to excessive ECM deposition and basement-membrane fibrosis. Symbol legend: ‘→’ indicates activation/promotion; ‘┤’ indicates inhibition/negative feedback; ‘↔’ indicates synergy; ‘
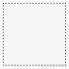
‘ indicates subordination.

In the TGF-β1 signaling pathway (mechanism detailed in **Figure 2**), TGF-β1 binds to its receptors (TβRII/TβRI) and induces receptor oligomerization (denoted as ‘Dimer’ in **Figure 2**). This subsequently activates the Smad2/3-Smad4 complex, which translocates to the nucleus to regulate epithelial cell-related genes (maintaining TJs or inducing EMT in the context of fibrosis). This pathway exhibits bidirectional regulatory effects in various diseases related to mucosal barriers (21, 22). Under normal conditions, this signaling pathway maintains the integrity of the mucosal barrier by preserving TJs between epithelial cells (such as ZO-1 and Claudin) and regulating cell differentiation. In pathological states, during the fibrotic process, this signaling promotes Epithelial-Mesenchymal Transition (EMT), leading to the loss of TJ proteins such as E-cadherin in epithelial cells, which acquire mesenchymal characteristics and weaken the mucosal barrier function (21, 23, 24). Notably, Smad7, as a core negative feedback factor, when downregulated, can relieve the inhibition of TGF-β signaling, exacerbating barrier damage. It is important to note that the aberrant activation of Smad1/5 induces EMT by phosphorylating Snail protein. Specifically, phosphorylated Snail protein acts as a transcription factor that directly binds to the promoter region of the E-cadherin gene, inhibiting its expression, leading to the loss of intercellular adhesion in epithelial cells and the acquisition of mesenchymal characteristics, thereby weakening barrier function, while Smad2/3 primarily regulates the expression of TJ proteins (23, 24).

**Figure 3:**
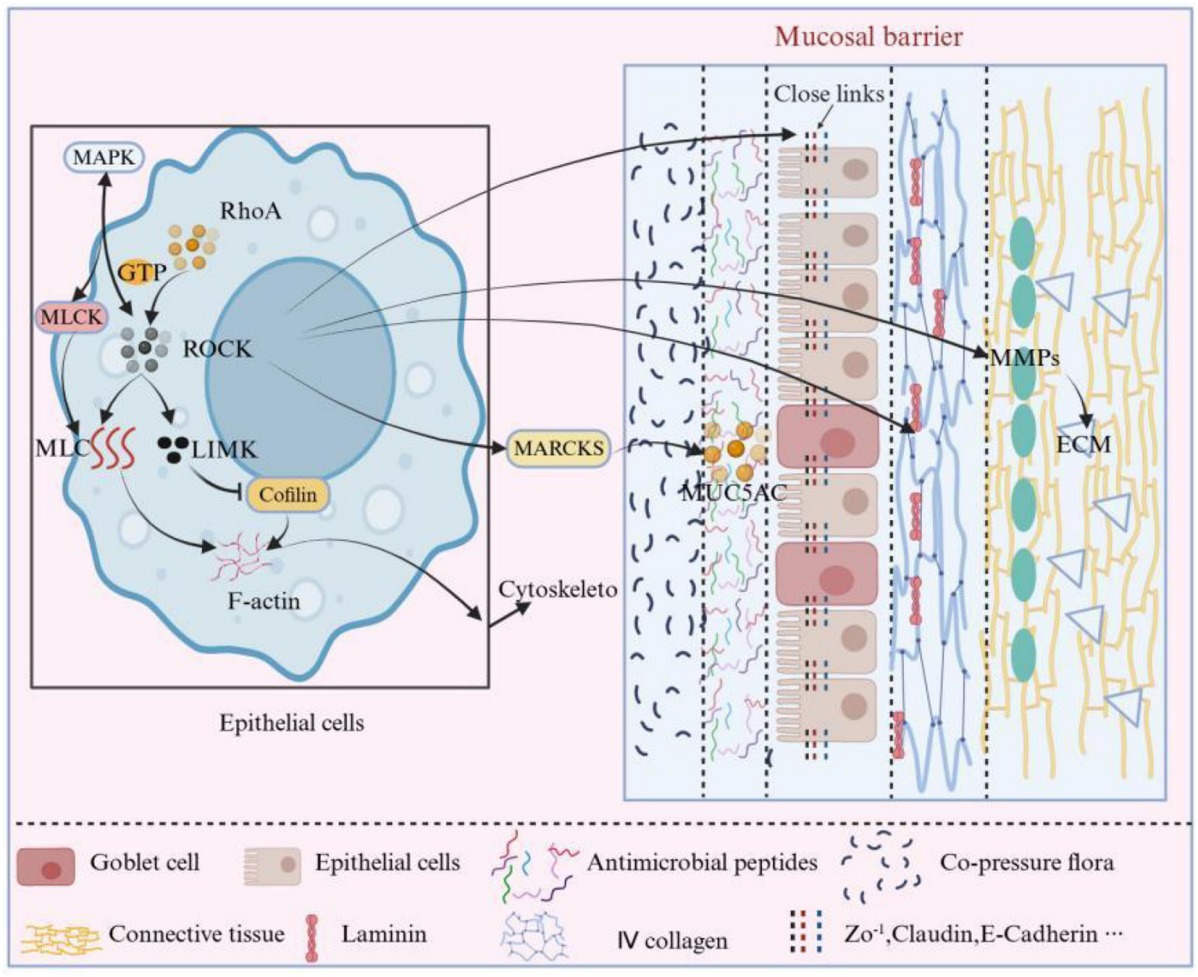
RhoA/ROCK signaling pathway and its regulation of mucosal barrier components. This diagram shows RhoA-GTP–mediated activation of ROCK and its key downstream effectors. ROCK phosphorylates MLC and cooperates with MLCK to drive actin–myosin contraction and cytoskeletal remodeling. It further acts through LIMK–Cofilin to regulate F-actin dynamics, and through MARCKS to promote MUC5AC-containing granule transport. ROCK also modulates MMPs and ECM components (type IV collagen, laminin), integrating with MAPK signaling at the MLCK node to coordinate mechanical tension, secretion and basement-membrane remodeling. Key regulatory points (1) Epithelial cells: RhoA/ROCK regulates cell morphology, contractility and migration via actin–myosin and LIMK–Cofilin pathways, participating in epithelial repair and restitution. (2) TJs: Through MLC phosphorylation and actin rearrangement, RhoA/ROCK controls the localization and expression of ZO-1, Claudins and Occludin; under inflammatory conditions, excessive activation leads to tight-junction reduction or relocalization and increased permeability. (3) Mucus layer: The ROCK–MARCKS axis promotes mucin granule transport and secretion (especially MUC5AC in airway epithelium), thereby affecting mucus-layer thickness and defensive capacity. (4) Basement membrane/ECM: ROCK regulates the secretion and reconstruction of ECM components (type IV collagen, laminin) and, when overactivated, induces MMPs (such as MMP-9), resulting in enhanced basement-membrane degradation and barrier weakening. Symbol legend: ‘→’ indicates activation/promotion; ‘┤’ indicates inhibition/negative feedback; ‘↔’ indicates synergy; ‘
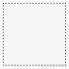
‘ indicates subordination.

The RhoA/ROCK signaling pathway (mechanism detailed in **Figure 3**) plays a crucial role in epithelial barrier repair by regulating cell morphology, contractility, migratory capacity, and the restoration of intercellular junctions. Upon ROCK activation, myosin light chain (MLC) is phosphorylated, and this process is synergistically regulated with the actin polymerization through the shared effector molecule myosin light chain kinase (MLCK) in the MAPK pathway. ROCK phosphorylates LIMK (which belongs to the LIM kinase family and is primarily responsible for the phosphorylation and regulation of actin-binding proteins to control cell morphology and movement). The activated LIMK subsequently phosphorylates Cofilin (a key actin depolymerizing factor that can sever and depolymerize actin filaments, thereby affecting the reorganization of the cytoskeleton), significantly impacting the function of epithelial cells (25, 26). Under inflammatory conditions, RhoA/ROCK activation can enhance ERK phosphorylation, forming a positive feedback loop that exacerbates barrier damage. MLCK, as a downstream hub of RhoA and MAPK, is responsible for regulating MLC phosphorylation and the relocalization of TJ proteins. Overexpression models of MLCK demonstrate an accelerated relocalization of TJ proteins (such as the internalization of ZO-1 from the membrane junction), which is validated through co-immunoprecipitation experiments (27, 28). However, excessive activation of this pathway may lead to excessive cell contraction, abnormal migration, or barrier damage. Therefore, the fine regulation of its activity is crucial for maintaining the integrity of the epithelial barrier (29).

**Figure 4:**
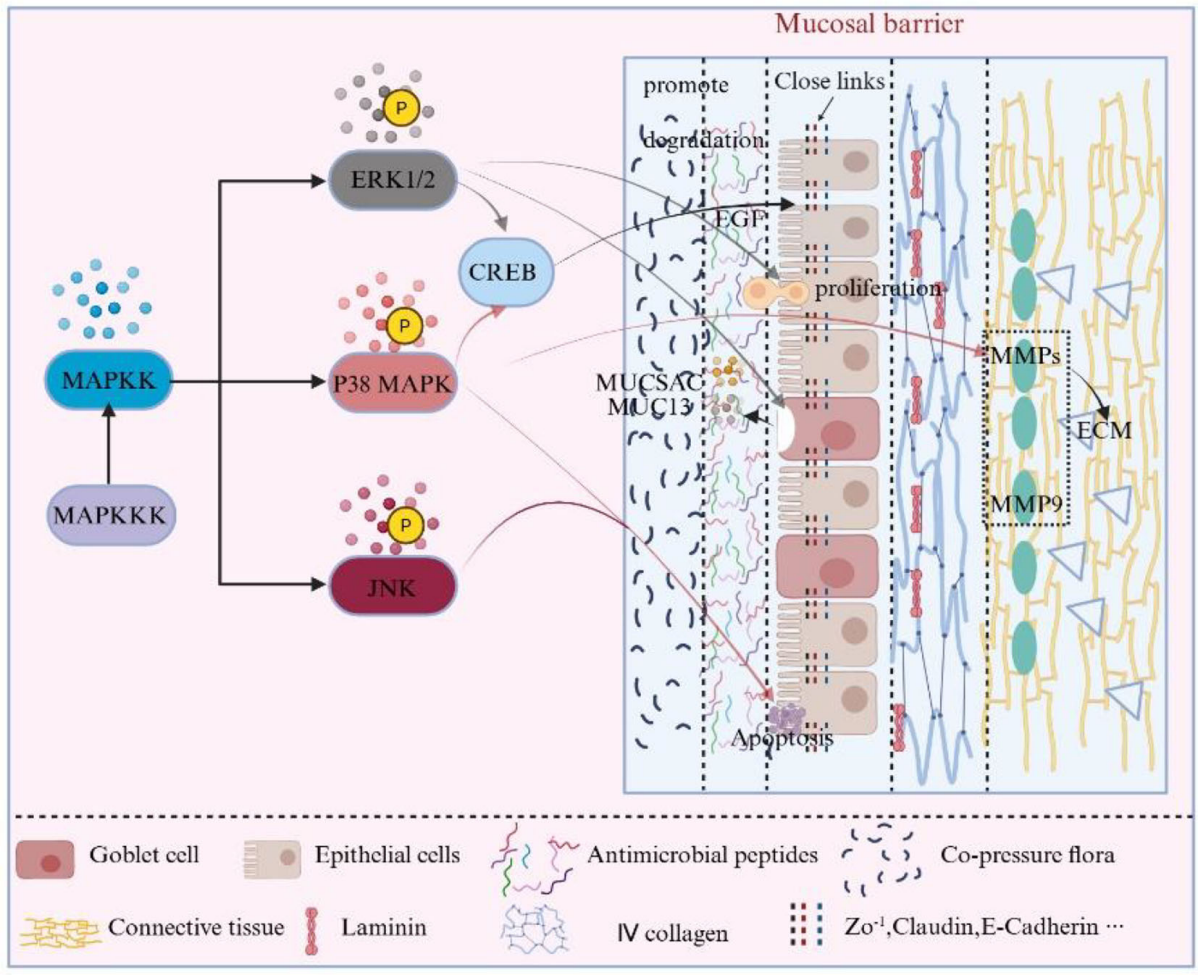
MAPK cascade and its regulation of mucosal barrier components. The MAPK kinase cascade (MAPKKK → MAPKK → MAPK) with ERK1/2, p38 and JNK as terminal branches,highlights phosphorylation-dependent activation steps and shows how each branch regulates epithelial proliferation and stress responses, tight-junction protein expression via CREB, mucin gene expression (MUC5AC/MUC13) and basement-membrane remodeling through MMPs and ECM components. Key regulatory points (1) Epithelial cells: ERK1/2 promotes epithelial proliferation and repair, whereas p38 and JNK sense stress and regulate differentiation and apoptosis; excessive p38 activation can induce ER-stress-related apoptosis and disrupt barrier integrity. (2) TJs: ERK1/2 and p38 branches increase ZO-1 and Occludin expression via CREB phosphorylation, enhancing paracellular sealing; in IBD, abnormal MAPK activation is associated with decreased Claudin-1, weakened TJs and increased permeability. (3) Mucus layer: MAPK signaling upregulates MUC5AC and MUC13 and affects goblet-cell differentiation; in the airway, activation of MAPK enhances MUC5AC expression and forms a protective mucus barrier, while overactivation causes mucus hypersecretion and airway obstruction; in the gut, ERK1/2 inhibition decreases MUC13 expression and can slow inflammation-induced erosion of the mucosal barrier. (4) Basement membrane/ECM: p38 MAPK upregulates MMP-9 to promote degradation and remodeling of basement-membrane components (e.g. laminin and type IV collagen), while ERK1/2 facilitates fibronectin expression and basement-membrane reconstruction; dysregulated activation may result in excessive degradation or maladaptive remodeling. Symbol legend: ‘→’ indicates activation/promotion; ‘┤’ indicates inhibition/negative feedback; ‘↔’ indicates synergy; ‘
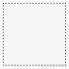
‘ indicates subordination; “●” symbol indicates phosphorylation sites/kinase activation events.

The MAPK signaling pathway (detailed mechanism in **Figure 4**) maintains the homeostasis of the mucosal barrier by regulating the proliferation, differentiation, and apoptosis of epithelial cells. The ERK1/2 branch plays a crucial role in promoting epithelial cell proliferation; excessive activation of ERK1/2 can accelerate cell turnover through the enhancement of Epidermal Growth Factor signaling, thereby maintaining barrier repair capacity, but it may also lead to abnormal cell proliferation (30). Additionally, p38 MAPK、JNK regulates epithelial cell differentiation and induces apoptosis by sensing stress signals, helping to eliminate damaged or mutated cells to ensure tissue integrity. Inhibition of p38 MAPK can prolong the lifespan of stem cells and improve epithelial repair efficiency (31, 32).

**Figure 5:**
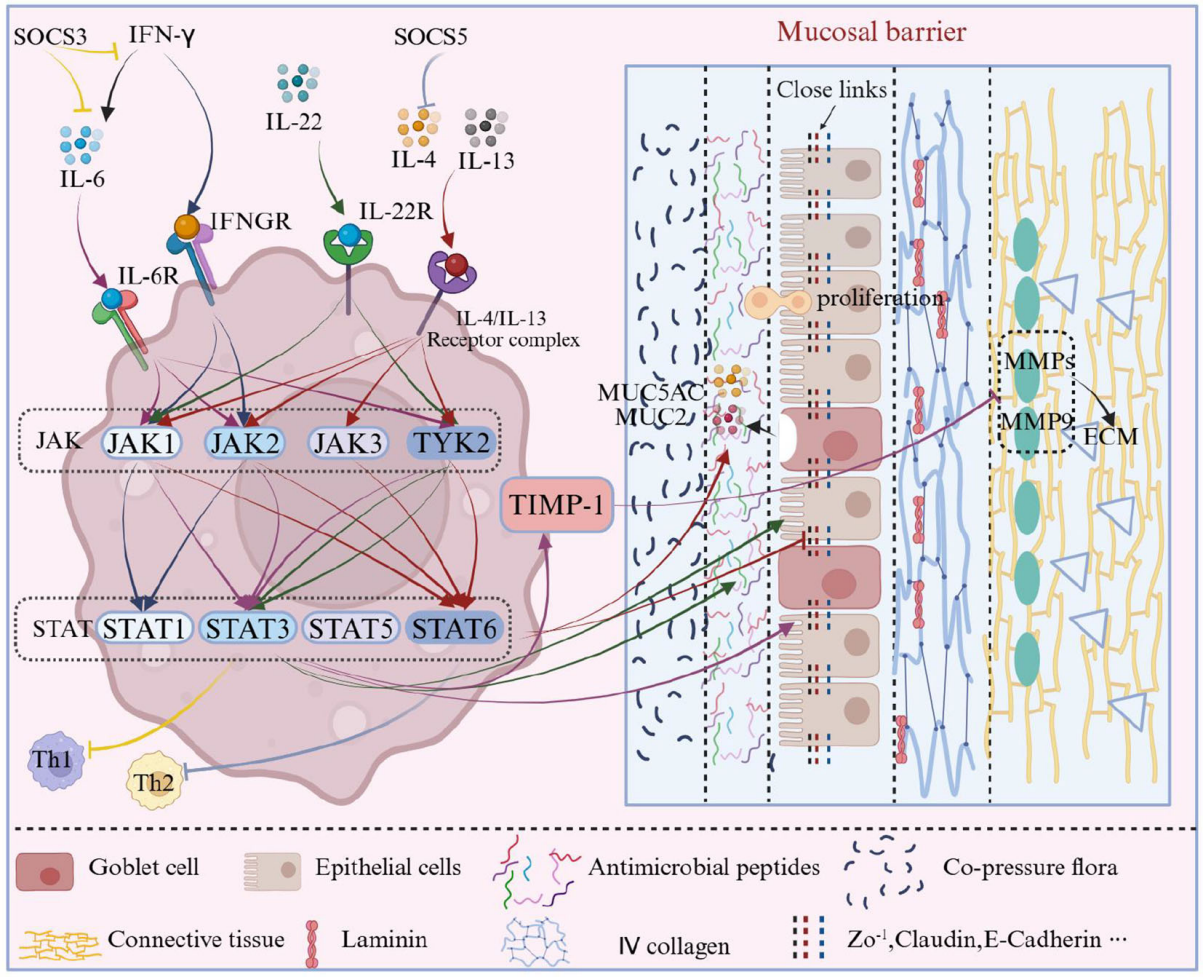
JAK/STAT signaling pathway and its regulation of mucosal barrier components. This figure provides an overview of cytokine-driven JAK/STAT signaling in the mucosal barrier. At the top, representative cytokines (such as IL-4, IL-6, IL-13, IL-22 and IFN-γ) and their receptors are shown. In the middle, the receptor–JAK–STAT cascade is mapped (for example IL-6/IL-22–JAK1/JAK2/TYK2–STAT3, IL-4/IL-13–JAK–STAT6, IFN-γ–JAK1/JAK2–STAT1). At the bottom, STAT1/3/5/6 outputs are summarized, including epithelial proliferation and repair, regulation of tight-junction proteins, control of mucin genes (MUC5AC/MUC2), and basement-membrane remodeling via MMP-2/MMP-9 and ECM proteins. The SOCS family is indicated as a negative-feedback module that restrains excessive pathway activation. Key regulatory points (1) Epithelial cells: The IL-6/IL-22–STAT3 axis promotes epithelial proliferation, damage repair and barrier restitution; sustained overactivation, however, can amplify inflammation and disturb homeostasis. (2) TJs: JAK/STAT signaling maintains intercellular connections by upregulating claudins and ZO-1 (for example IL-6–STAT3–claudin-1/claudin-4); conversely, excessive IL-4/IL-13–STAT6 activation under allergic-type inflammation disrupts TJs and increases permeability. (3) Mucus layer: IL-4/IL-13–STAT6 directly binds to the promoters of MUC5AC and MUC2 and, together with co-activators such as CBP/p300, enhances transcription and mucus secretion; STAT3, activated by IL-6/IL-22, regulates epithelial secretion of antimicrobial proteins and repair factors, strengthening mucus-layer barrier function. Overactivation can cause mucus hypersecretion or an imbalanced mucus layer. (4) Basement membrane/ECM: STAT3-dependent regulation of MMP-2/MMP-9 and ECM proteins (fibronectin, laminin, type IV collagen) exerts a dual effect on basement-membrane degradation and repair. Disturbance of the STAT3-TIMP-1/MMP-9 dynamic equilibrium (for example excessive STAT3 phosphorylation) favors MMP-dominated basement-membrane degradation, leading to structural weakening and disease progression. Symbol legend: ‘→’ indicates activation/promotion; ‘┤’ indicates inhibition/negative feedback; ‘↔’ indicates synergy; ‘
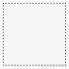
‘ indicates subordination; different colors represent different pathway mappings and functions.

The JAK-STAT signaling pathway (mechanism detailed in **Figure 5**) plays a central role in maintaining the regenerative function of epithelial cells and repairing barrier damage by regulating their proliferation and differentiation. Multiple cytokines participate in epithelial regeneration and repair through specific JAK/STAT pathways. For instance, IL-6 and IL-22 primarily activate STAT3 through JAK1/TYK2 (or JAK1/JAK2/TYK2), promoting epithelial cell proliferation, the expression of TJ proteins, and tissue repair (33–35). IFN-γ mainly activates STAT1 via JAK1/JAK2, exerting immunoregulatory and anti-pathogen functions, with its primary biological effects mediated by STAT1 rather than directly through STAT3. Notably, the IFN-γ-related STAT3 activation observed in inflammatory environments is often an indirect effect, typically dependent on secondary cytokines induced by IFN-γ (e.g., IL-6) (36–38). The Suppressor of Cytokine Signaling (SOCS) family regulates the JAK–STAT pathway through negative feedback to maintain intracellular homeostasis; however, the roles of different members in immune regulation are member-specific. Specifically, SOCS5 selectively inhibits the IL-4/STAT6-mediated Th2 differentiation pathway (39), while SOCS3 plays a significant negative regulatory role on the IL-6/STAT3 and IFN-γ/STAT1 axes, influencing Th1 responses (40, 41).

There is a cross-regulatory relationship between the TGF-β/Smad and Wnt/β-catenin pathways. Smad7, as a negative feedback factor in TGF-β signaling, can enhance Wnt activity by inhibiting the phosphorylation of TGF-βRI. Meanwhile, nuclear β-catenin forms a complex with Smad4, directly binding to the promoter region of the Snail gene to induce EMT. This mechanism significantly affects barrier repair efficiency in chronic inflammation models.

### 4.2 Regulation and influence on TJs

TJs are key components of the integrity of the mucosal barrier (42). They are primarily composed of transmembrane proteins such as claudins and occludins, as well as cytoskeletal proteins like ZO proteins. The core function of TJs is to form an almost impermeable physical barrier that prevents the invasion of pathogens, toxins, and undigested substances through the paracellular pathway into tissues. At the same time, they act as a sophisticated ‘molecular gate’, selectively allowing specific ions, water, and solutes to pass through the paracellular transport by regulating the composition and conformation of transmembrane proteins, thus facilitating material exchange and signal transduction. The integrity of their structure and the precise regulation of their function are crucial for effective barrier protection, and dysfunction in these processes is closely associated with the occurrence and development of various mucosal-related diseases, such as IBD, allergies, and infections.

The Wnt/β-catenin signaling pathway (mechanism detailed in **Figure 1**) ensures TJs between epithelial cells by regulating the expression of TJ proteins, preventing pathogens and harmful substances from entering the body through intercellular gaps (17). In the intestinal microenvironment, high expression of WNT2b (primarily derived from fibroblasts) can bind to the Frizzled receptors (such as FZD4) and co-receptors LRP5/6 on the surface of epithelial cells (Frizzled (FZD): Wnt receptors on the cell membrane; LRP5/6: single-channel co-receptors required for canonical Wnt signaling), forming a Wnt-Frizzled-LRP5/6 ternary complex that activates the classical Wnt/β-catenin signaling pathway (43, 44), thereby altering the expression and localization of TJ proteins (such as ZO-1 and E-cadherin) (45). The TJ proteins also have a dynamic feedback effect on the Wnt/β-catenin signaling pathway; in the nasal polyp tissues of patients with Chronic Rhinosinusitis with Nasal Polyps (CRSwNP), the activation of the Wnt/β-catenin signaling pathway is associated with a reduction in TJ proteins (such as E-cadherin), leading to the loss of intercellular connections and weakening the barrier function of the nasal mucosa (46).

The TGF-β/Smad signaling pathway (mechanism detailed in **Figure 2**) activates the expression of TJ proteins such as ZO-1, Occludin, and Claudin through the phosphorylation of Smad2 and Smad3, thereby maintaining the adhesion and barrier functions between epithelial cells. When this signaling pathway is excessively activated, as seen in diseases such as fibrosis and Chronic Rhinosinusitis (CRS), it can significantly reduce the levels of TJ proteins, leading to impaired barrier function, accompanied by the expression of fibrotic factors such as Connective Tissue Growth Factor (CTGF), which further disrupts the epithelial barrier (47–49). Conversely, when the expression of this signaling pathway is insufficient, as observed in IBD, the inhibition of the TGF-β/Smad signaling leads to decreased levels of TJ proteins like ZO-1 and Claudin, compromising the integrity of the epithelial barrier and increasing intestinal permeability, thus exacerbating inflammation and pathological damage (50, 51).

The RhoA/ROCK signaling pathway (mechanism detailed in **Figure 3**) plays a crucial role in the damage and repair of mucosal barrier function by regulating the cytoskeleton and TJ proteins. Studies have found that the RhoA/ROCK signaling pathway maintains TJs between cells by promoting the phosphorylation of MLCs and the rearrangement of actin filaments, thus preventing the widening of intercellular spaces (52, 53). Under pathological conditions, inflammatory factors such as TNF-α, IL-6, and HMGB1 activate RhoA/ROCK, leading to a reduction and relocalization of TJ proteins (such as claudin-1, occludin, and ZO-1), thereby disrupting intercellular TJs and increasing barrier permeability (54).

TJs, as the core structure of the mucosal barrier, are directly regulated by the MAPK signaling pathway (mechanism details can be found in **Figure 4**). The p38 MAPK and ERK branches promote an increase in the expression of ZO-1 and Occludin through the phosphorylation of CREB, which affects the selective permeability between cells and enhances barrier function (30). However, excessive activation of the MAPK signaling pathway, particularly induced by pathogens or inflammatory factors, can compromise the integrity of TJs. For instance, in IBD, abnormal activation of the MAPK pathway leads to a decrease in Claudin-1 expression, resulting in weakened barrier function and increased permeability (55).

The JAK-STAT signaling pathway (mechanism detailed in **Figure 5**) maintains intercellular connections by regulating the expression of TJ proteins such as claudins and ZO-1. IL-6 enhances the gene expression of claudin-1 and claudin-4 via the STAT3 pathway, reducing paracellular permeability and thereby improving the mucosal barrier (36). Conversely, under inflammatory conditions, the excessive activation of the JAK-STAT pathway by IL-4 and IL-13 disrupts TJs and weakens barrier function (41).

### 4.3 Regulation and influence on mucus layer

The Wnt/β-catenin signaling pathway (mechanism detailed in **Figure 1**) regulates the mucous layer primarily by influencing the differentiation and function of mucus-secreting cells, such as goblet cells. The Wnt/β-catenin pathway promotes the differentiation of epithelial stem cells into goblet cells, which are a crucial cell type responsible for mucus secretion. Studies have shown that the activation of the Wnt/β-catenin signaling pathway can regulate the secretion levels of mucins (such as MUC2), ensuring the formation of a protective mucous layer on the mucosal surface. This mucous layer serves to protect epithelial cells from direct attack by harmful substances (56).

The TGF-β/Smad signaling pathway (mechanism detailed in **Figure 2**) plays a crucial regulatory role in the function of the mucus layer. Under physiological conditions, this pathway maintains the structural stability of epithelial cells and the ECM by upregulating components such as fibronectin and collagen. It promotes the proliferation and differentiation of epithelial cells through the phosphorylation of Smad3, indirectly supporting the function of goblet cells and ensuring the stability of mucus secretion. Under pathological conditions, abnormal activation of the TGF-β1 signaling pathway damages goblet cell function by enhancing the release of inflammatory factors (such as IL-1β and IL-6) and oxidative stress, leading to a reduction in mucus secretion. Moreover, TGF-β induces the expression of fibrosis-related genes (such as α-SMA), resulting in epithelial tissue fibrosis, which further weakens the protective function of the mucus layer. The interaction between TGF-β and the Wnt/β-catenin and Notch signaling pathways exacerbates these effects, potentially leading to dysfunction of the mucus layer and reduced protective capacity of the mucosal barrier under pathological conditions (57, 58).

The RhoA/ROCK signaling pathway (as detailed in **Figure 3**) plays a crucial role in mucus secretion by regulating the fluid transport and secretion functions of epithelial cells. Upon activation of ROCK, it promotes cell contraction through the phosphorylation of MLCs, enhancing the process of transporting secretory vesicles (such as mucin granules) to the cell surface and facilitating their fusion with the cell membrane, thereby increasing mucus secretion (59). Furthermore, ROCK also optimizes the stability and thickness of the mucus layer by influencing the reorganization of the cytoskeleton, adjusting intercellular junctions and the polarity of epithelial cells. This mechanism is particularly vital for the formation and protection of the mucus barrier in tissues with significant barrier functions, such as the gastrointestinal and respiratory tracts (60). However, due to tissue-specific differences, the weight of the RhoA/ROCK signaling pathway’s effects may vary. In the respiratory mucosa, the RhoA/ROCK pathway specifically promotes MUC5AC secretion by phosphorylating Myristoylated Alanine-Rich C-Kinase Substrate(MARCKS), a mechanism that has been shown to enhance the defensive function of the mucus layer in asthma models (61); whereas in the intestine, this pathway primarily regulates cell migration rather than mucus secretion (62).

The MAPK signaling pathway (mechanism detailed in **Figure 4**) influences the thickness and function of the mucus layer on mucosal surfaces by regulating the expression of mucus-related genes such as MUC5AC and MUC13. In respiratory diseases, MAPK activation can enhance the expression of MUC5AC, forming a protective barrier against external pathogens. However, under pathological conditions (such as asthma and COPD), excessive activation of the MAPK pathway can lead to excessive mucus secretion, exacerbating airway obstruction and inflammatory responses (63). In gastrointestinal diseases, the MAPK signaling pathway is closely related to the differentiation of mucus-secreting cells, such as goblet cells. Studies have shown that inhibiting ERK1/2 can reduce the expression of MUC13, thereby slowing the erosion of the mucosal barrier caused by inflammation (64).

The JAK-STAT signaling pathway (mechanism detailed in **Figure 5**) plays a crucial role in the generation and function of the mucus layer by regulating the signal transduction of cytokines such as IL-4, IL-6, and IL-13. Under stimulation by IL-4 and IL-13, STAT6 translocates into the nucleus and directly binds to the promoter regions of the MUC5AC and MUC2 genes, enhancing transcription by recruiting co-activators such as CBP/p300, thereby promoting mucus secretion. Conversely, STAT3 regulates the secretion of antimicrobial proteins and repair factors by epithelial cells under the influence of IL-6 and IL-22, enhancing the barrier function of the mucus layer, which is particularly evident in asthma and allergic diseases (65). However, excessive activation of this pathway may lead to an imbalance in mucus secretion, such as excessive mucus accumulation in COPD, exacerbating airway obstruction (66); in IBD, the abnormal mucus layer weakens the intestinal barrier and increases susceptibility to pathogen invasion (67). The JAK-STAT pathway is not only a key regulatory factor in maintaining the mucus layer but also a core mechanism underlying its pathological changes, making targeting this pathway a promising therapeutic approach for related diseases.

### 4.4 Effect on basement membrane

The basement membrane is composed of ECM, and its integrity is crucial for the structure and function of the mucosal barrier. The Wnt/β-catenin signaling pathway (mechanism detailed in **Figure 1**) maintains the structure and function of the basement membrane by regulating MMPs and ECM components. The Wnt/β-catenin signaling can modulate the expression of MMPs, which are enzymes responsible for the remodeling and repair of the basement membrane. When the basement membrane is damaged, MMPs degrade the damaged components of the basement membrane, while components such as fibronectin, regulated by the Wnt signaling pathway, promote the repair and regeneration of the basement membrane (68). Additionally, the Wnt/β-catenin pathway can maintain the balance of ECM components of the basement membrane, such as by regulating the expression of type IV collagen, ensuring that the basement membrane has appropriate structural strength and functional integrity (69, 70).

The TGF-β/Smad signaling pathway (mechanism detailed in **Figure 2**) maintains the strength and function of the basement membrane by regulating the production of ECM components such as collagen and fibronectin. Moderate activation of this pathway promotes tissue repair (e.g., MMP-mediated remodeling), while persistent overactivation can lead to pathological fibrosis, resulting in structural destruction and functional loss of the basement membrane. For instance, in diseases such as liver fibrosis and kidney fibrosis, excessive activation of this pathway causes an overaccumulation of ECM, disrupting the normal structure and function of the basement membrane, making the mucosal barrier more susceptible to damage (21).

The RhoA/ROCK signaling pathway (mechanism detailed in **Figure 3**) significantly influences the structure and function of the basement membrane by regulating the cytoskeleton, cell polarity, and ECM metabolism. This pathway enhances the contractile force of actin-myosin fibers, maintaining mechanical stability between cells and the basement membrane, while also regulating the secretion of ECM components such as type IV collagen and laminin (71). Under pathological conditions, RhoA/ROCK activates MMPs, promoting the degradation of the basement membrane and weakening its barrier function, thereby facilitating tumor invasion (72, 73). Furthermore, this pathway maintains epithelial cell polarity, preventing barrier damage caused by polarity disruption, and promotes fibroblast migration and ECM reconstruction during injury repair, restoring the integrity of the basement membrane.

The MAPK signaling pathway (mechanism detailed in **Figure 4**) participates in the dynamic repair and remodeling of the basement membrane by regulating the expression of MMPs (such as MMP9) and ECM components. Under pathological conditions, the activation of p38 MAPK enhances the expression of MMP-9 through the regulation of transcription factors, promoting the degradation of damaged basement membrane components. Meanwhile, the ERK pathway facilitates basement membrane remodeling and accelerates repair by enhancing the expression of FN. p38 MAPK may indirectly participate in the repair process by cross-regulating the ERK pathway (74).

The JAK-STAT signaling pathway (mechanism detailed in **Figure 5**) exerts a dual effect on the degradation and repair of the basement membrane by regulating the signaling of inflammatory factors. Under inflammatory conditions, IL-6 can activate STAT3, while IFN-γ can indirectly promote the activation of STAT3 by inducing secondary cytokines (such as IL-6) or through signal crosstalk. This upregulates MMP-2 and MMP-9, facilitating the degradation of basement membrane components such as type IV collagen, thereby weakening barrier function, which is particularly significant in tumor invasion and chronic inflammation (75, 76). Concurrently, STAT3 promotes basement membrane reconstruction and tissue repair by regulating the expression of fibronectin and laminin. An imbalanced JAK-STAT pathway can result in abnormal thickening of the basement membrane or impaired repair. For instance, under inflammatory conditions, after activation of STAT3 through the Ser727 phosphorylation site, it binds to the promoter region of MMP-9 to enhance its transcription, while also interacting with the STAT response element of the TIMP-1 gene, forming a dynamic equilibrium network. When this balance is disrupted (e.g., excessive phosphorylation of STAT3), it leads to an exacerbation of MMP-9 dominant basement membrane degradation, compromising barrier structure and worsening disease progression (77). In inflammatory models, the Tyr705 phosphorylation of STAT3 can be induced by IL-6 and is regulated by a JAK1-mediated feedback loop that adjusts the TIMP-1/MMP-9 ratio, which is crucial for basement membrane repair.

### 4.5 Synergistic and antagonistic networks of key pathways

There exists a dynamic interplay among various signaling pathways within the mucosal barrier, collectively shaping the onset and progression of diseases (**Table 2**). Moreover, the “weight” of each pathway varies across different diseases.

**Table 2:** Disease-specific dominant signaling axes and pathway crosstalk in major mucosal barrier disorders.

Diseases	Dominant signals specific to diseases	Key signal interactions within the signaling network	Outcomes related to the mucosal barrier
Asthma	IL-4/IL-13 → JAK-STAT6; TGF-β → Smad2/3 TGF-β → TAK1/p38-JNK → WNT5A	STAT6↑ → Th2 shift/IgE↑; TGF-β/Smad2/3↑ → EMT/fibrosis; TGF-β→WNT5A↑→Ca^2+^/JNK–NFAT → ECM/α-SMA; upstream cytokines → RhoA/ROCK, MAPK (effector arms)	Type-2 inflammation↑, mucus hypersecretion↑, airway hyper-reactivity↑, epithelial repair–EMT imbalance, basement-membrane thickening/airway remodelling↑.
IBD	Wnt/β-catenin (ISC renewal/repair); JAK-STAT (IL-6/IL-22 axis)	Wnt ⟷ JAK-STAT (context-dependent antagonism/synergy): Wnt↑ → regeneration/TJ stability↑; sustained JAK-STAT↑ ⊣ TJ → permeability↑ & inflammation amplification	Repair ↔ chronic leakiness balance (set by Wnt vs JAK-STAT “weights”).
Chronic Sinusitis	TGF-β/Smad2/3; Wnt/β-catenin (EMT axis)	TGF-β/Smad + Wnt/β-catenin → EMT↑, myofibroblast activation; S100A4/PP2A → Wnt activation; MAPK/NF-κB↑ amplifies inflammation-to-remodelling coupling	Epithelial integrity↓, persistent EMT, basement-membrane thickening, polyp growth.

“→” activation/flow; “⊣” inhibition; “⟷” bidirectional; “+” synergy; “↑/↓” increase/decrease.

From the perspective of disease phenotypes, the aforementioned signaling networks often exhibit distinct ‘dominant’ pathways among different mucosal barrier diseases.

In asthma, a substantial body of basic research and clinical evidence indicates that the IL-4/IL-13-JAK-STAT6 axis plays a pivotal upstream role, driving Th2 cell differentiation, class switching to IgE, excessive mucus secretion, and the recruitment of eosinophils (78–80). Meanwhile, the TGF-β/Smad2/3 signaling pathway acts as a primary mediator involved in airway remodeling and submucosal fibrosis (81). Wnt5a also acts as an important interaction hub for TGF-β’s role in asthma; TGF-β can induce the expression of WNT5A in airway smooth muscle cells through transcriptional regulatory pathways such as TAK1–p38/JNK and β-catenin/Sp1, thereby promoting ECM production and enhancing α-SMA expression via non-classical signaling pathways like Ca^2+^/JNK–NFAT, which leads to fibrotic airway remodeling (82, 83). An interesting finding in epithelial cell models is that physiological levels of WNT5A can, on one hand, promote wound healing, while on the other, induce morphological changes similar to EMT, suggesting a bidirectional regulatory role (84, 85). As for the RhoA/ROCK and MAPK pathways, they primarily serve as downstream effector arms, translating mechanical stretch and upstream cytokine signals into smooth muscle contraction, goblet cell metaplasia, and epithelial stress responses (78, 86).

In IBD, The Wnt/β-catenin–JAK-STAT epithelial axis plays a crucial role in maintaining intestinal epithelial stem cell renewal and crypt homeostasis through classical Wnt/β-catenin signaling (18, 87, 88). There exists a bidirectional interaction between Wnt/β-catenin and JAK-STAT: Wnt signaling promotes epithelial regeneration and barrier repair, while persistent activation of JAK-STAT shifts the balance towards chronic inflammation and barrier failure (36, 89). The excessive activation of JAK-STAT mediated by IL-6, IL-22, and others disrupts TJs, increases epithelial permeability, and amplifies mucosal inflammation (33, 36, 90). Consequently, the abnormal Wnt-JAK-STAT interaction becomes a critical dominant axis driving the transition of the intestinal barrier from a repair state to chronic barrier failure and microbial dysbiosis.

In chronic sinusitis, The Wnt/β-catenin and TGF-β/Smad-dependent pathways, as well as the Wnt/β-catenin-related EMT axis—TGF-β/Smad2/3 and Wnt/β-catenin—are the primary signaling pathways that regulate EMT, collagen deposition, and basement membrane thickening (91–94). Smad7 inhibits the enhancement of Wnt activity by TGF-β (94). The TGF-β/Smad pathway collaborates with Wnt/β-catenin and upstream mediators such as S100A4 and PP2A to induce EMT, myofibroblast activation, and tissue remodeling (95, 96). Additionally, pathways such as MAPK and NF-κB further regulate the EMT program, tightly linking (97).

### 4.6 Synergistic and antagonistic effects of other signaling pathways

**Figure 6:**
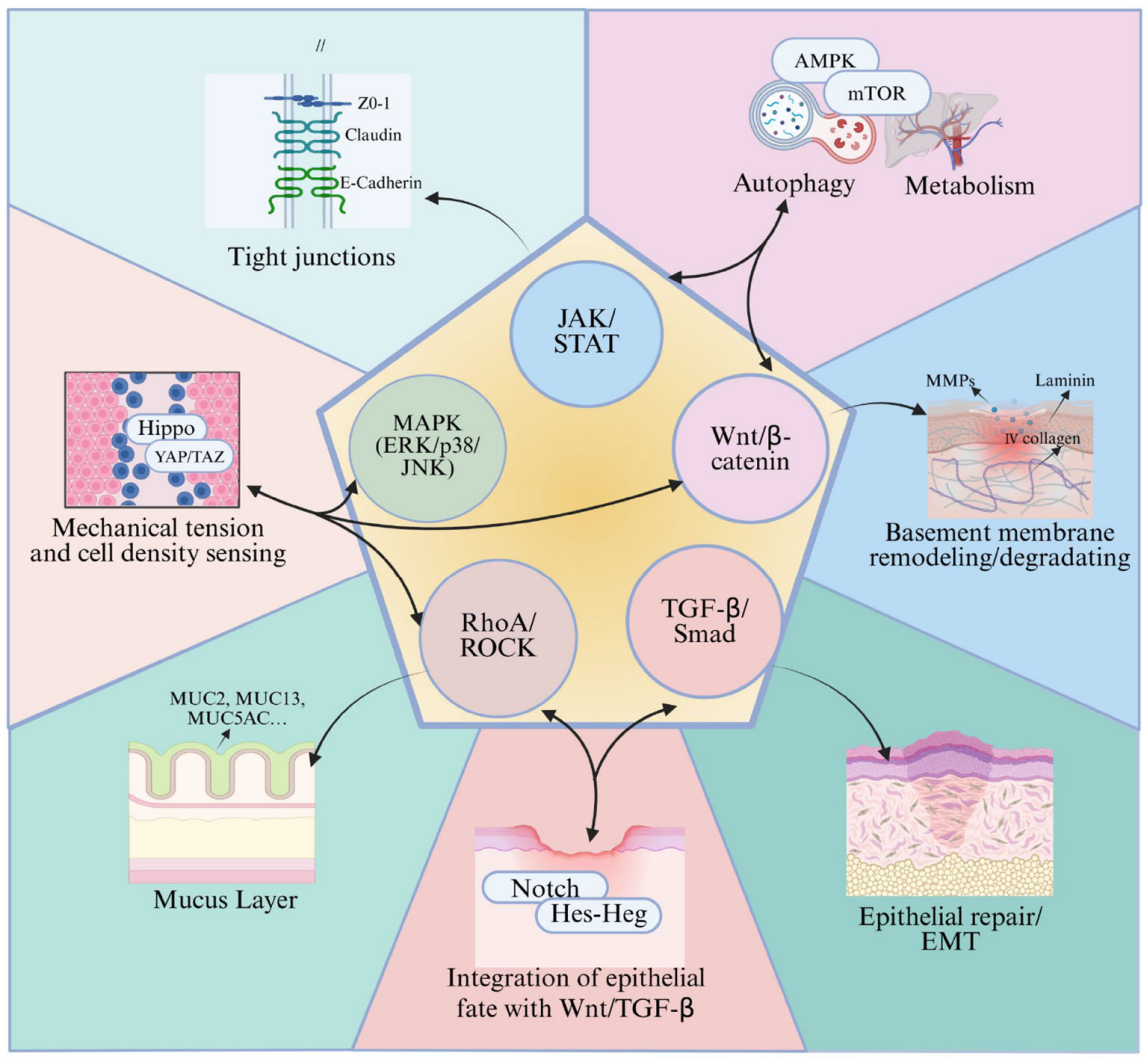
Schematic diagram of the integrative interaction network of five core signaling pathways of the mucosal barrier with Notch/Hes–Hey, Hippo, and AMPK–mTOR.

In addition to the five core pathways, some upstream or parallel signaling modules further refine the response of the mucosal barrier (**Figure 6**).

The Notch pathway and its downstream Hes/Hey gene family are regarded as a transcriptional “crossroad” between Wnt/β-catenin and TGF-β/Smad signaling (98, 99). Notch-Hes1 can integrate Wnt signals at the promoter level, dynamically balancing the self-renewal and differentiation of intestinal epithelial stem cells, thereby preventing abnormal proliferation caused by excessive Wnt activation and avoiding stem cell depletion due to excessive inhibition. This balance plays a crucial role in maintaining the ratio of absorptive cells to goblet cells and ensuring epithelial integrity.

The Hippo-YAP/TAZ pathway, by sensing various microenvironmental factors such as cytoskeletal tension, cell density, and inflammatory mediators, is highly coupled with RhoA/ROCK-mediated actin remodeling and the MAPK and TGF-β/Smad cascades (100). Activation of YAP/TAZ can expand the intestinal stem cell population and promote epithelial regeneration after mucosal injury (101). Additionally, through interactions with TJ-associated proteins such as angiomotin, YAP/TAZ regulates TJ structure and barrier permeability. In airway epithelium, YAP/TAZ also participates in limiting the excessive differentiation of goblet cells, maintaining the homeostasis of the mucus layer, which is of significant importance in diseases such as asthma and chronic rhinosinusitis (102).

The AMPK–mTOR, as a metabolic “master switch,” transmits signals from nutrient status, growth factors, and inflammatory cytokines through pathways such as MAPK and JAK/STAT to downstream targets, primarily by inhibiting autophagy via mTORC1 (103). This regulation affects the degradation and recycling of TJ proteins, mitochondrial function, as well as the secretion of antimicrobial peptides and mucus (104). The AMPK–mTOR-dependent autophagy exhibits a context-dependent bidirectional effect on the epithelial barrier in infection and colitis models: moderate autophagy can help clear damaged organelles and protect TJs, while sustained mTOR overactivation or autophagy deficiency exacerbates epithelial cell apoptosis, mucus layer disruption, and increased intestinal permeability (104, 105).

Overall, Notch/Hes–Hey, Hippo, and mTOR/autophagy are not isolated modules but rather embedded as “regulatory units” within five core pathways, serving to sense multidimensional signals such as mechanical, metabolic, and inflammatory inputs, and converting these inputs into fine-tuned regulation of epithelial repair, TJs, and mucus layer functions of the mucosal barrier.

## 5 Applications and strategies for the regulation of each signaling pathway

The abnormal activation or inhibition of signaling pathways is closely related to various mucosal-associated diseases. Targeting the regulation of these pathways may provide new strategies for the treatment of mucosal-associated diseases.

### 5.1 Pathway regulation strategies of mucosal barrier injury repair

The Wnt/β-catenin signaling pathway promotes epithelial cell repair and enhances TJs, effectively restoring damaged mucosal barriers and reducing barrier permeability. It can regulate inflammation and immune responses, playing a role in inhibiting excessive inflammatory reactions and restoring immune balance, making it a potentially important target for the treatment of inflammatory diseases and immune disorders (106). In response to mucosal barrier damage, regulating signaling pathways such as activating Wnt/β-catenin to promote epithelial repair and inhibiting RhoA/ROCK to alleviate permeability can serve as a universal repair strategy.

Regulating the TGF-β/Smad signaling pathway, lowering the levels of pro-inflammatory factors, or restoring immune balance can alleviate diseases caused by barrier damage. Genetic variations in the TGF-β signaling pathway (such as SMAD3’s rs4147358 and SMAD7’s rs12956924) affect the function of Smad proteins, thereby influencing intestinal immune balance and the inflammatory response (107, 108).

Regulating the RhoA/ROCK signaling pathway is an important strategy for repairing damaged mucosal barriers. By inhibiting the excessive activity of this pathway, it is possible to alleviate the increased barrier permeability caused by excessive contraction of the cytoskeleton, thereby restoring barrier function. The stability of the endothelial barrier depends on the balance of activity between RhoA (which promotes contraction) and its antagonistic molecule Rac1 (which maintains adhesion). In an inflammatory state, the elevated RhoA/Rac1 ratio leads to barrier disruption. M.Y. Radeva et al (109) found that under healthy conditions, the precise regulation of the activities of both molecules maintains barrier function; however, during inflammation, inflammatory mediators trigger an increase in RhoA activity and a decrease in Rac1 activity, resulting in the loss of barrier function.

Inhibiting the excessive activation of the MAPK signaling pathway is one of the effective strategies for restoring mucosal barrier function. The excessive activation of the p38/MAPK signaling pathway, characterized by a significant increase in p38 phosphorylation levels, triggers an endoplasmic reticulum stress response, upregulates the expression of pro-apoptotic proteins CHOP/TRIB3, induces apoptosis in gastric epithelial cells, and disrupts TJ proteins (Occludin, ZO-1), ultimately damaging the gastric mucosal barrier function. However, by specifically inhibiting p38/MAPK phosphorylation through the overexpression of MKP-5, it is possible to effectively reverse the aforementioned pathological processes—significantly reducing cell apoptosis, repairing TJ structures, and restoring the integrity of the mucosal barrier (110).

The JAK-STAT signaling pathway plays a crucial role in maintaining mucosal barrier function and immune homeostasis by regulating the expression balance of cytokines in inflammatory and immune dysregulation-related diseases. Optimizing the function of the JAK-STAT signaling pathway in response to its abnormal regulation has emerged as an effective strategy for treating diseases associated with mucosal barrier damage. Targeted interventions on the JAK-STAT pathway have demonstrated significant efficacy across various diseases. For instance, miR-375 alleviates mucosal damage in AR by inhibiting JAK2 and reducing the levels of inflammatory factors (111). In atopic dermatitis, JAK inhibitors mitigate chronic inflammation mediated by Th2-type cytokines, thereby alleviating symptoms (41, 112).

### 5.2 Regulation of allergic lesions

The Wnt/β-catenin signaling pathway has potential application value in the treatment of allergic diseases, particularly in alleviating reactions by modulating immune responses and improving barrier function. Li et al (113) found that by regulating the Wnt/β-catenin signaling pathway, the Th2-type immune response can be weakened, which helps to reduce the levels of inflammation in allergic diseases such as asthma, AR, and food allergies. Studies have shown that modulating this pathway may inhibit the production of IL-4, IL-5, and IL-13 under specific conditions, thereby reducing IgE-mediated type I hypersensitivity. On the other hand, activating the Wnt/β-catenin signaling pathway can promote the repair of epithelial cells and the restoration of TJs, improving mucosal barrier function. Enhancing the defensive capacity of the mucosal barrier can also reduce the penetration of allergens, thereby decreasing the incidence of allergic reactions.

TGF-β1 plays a crucial role in the repair of mucosa, particularly by regulating the proliferation and repair of epithelial cells through the phosphorylation of Smad3 (80, 114). However, its high expression in AR is closely associated with impaired mucosal barrier function, leading to increased apoptosis and elevated levels of inflammatory factors such as IL-4, IL-5, IL-17, and IFN-γ. Meanwhile, Smad7, as an inhibitor of TGF-β signaling, reduces TGF-β-induced inflammation and apoptosis by blocking signal transduction, but its decreased expression in AR further exacerbates barrier damage.

The RhoA/ROCK signaling pathway significantly influences the progression of allergic diseases by regulating cytoskeletal dynamics, epithelial barrier function, and the secretion of inflammatory factors. In asthma, the RhoA/ROCK pathway promotes airway smooth muscle contraction, airway hyperresponsiveness, and remodeling, while also regulating the differentiation of Th2 and Th17 cells, enhancing the secretion of pro-inflammatory factors, and exacerbating airway inflammation and structural abnormalities (115). In AR, the activation of this pathway disrupts nasal epithelial barrier function by downregulating TJ proteins (such as ZO-1) and increasing permeability, while also enhancing the secretion of TGF-β1 from airway epithelial cells and activating its downstream Smad3 signaling. This facilitates the easier penetration of allergens and triggers immune responses (116, 117). Targeting ROCK inhibitors (such as Fasudil and Y-27632) can significantly alleviate disease symptoms by inhibiting smooth muscle contraction and reducing the secretion of inflammatory factors (e.g., IL-4, IL-13). However, the efficacy of Fasudil has primarily been validated in animal models, and its clinical application needs to further address issues of tissue selectivity.

In allergic diseases, the MAPK signaling pathway mediates immune responses regulated by IL-33 and other inflammatory factors, participating in the pathophysiological processes. Inhibition of JNK signaling can reduce the activity of eosinophils, thereby alleviating symptoms of AR (118). The various components of apricot kernel gel can directly or indirectly activate the ERK signaling pathway, affecting the expression of DNA methyltransferases, and reducing the methylation in the promoter region of the IFN-γ gene, which contributes to the improvement of AR symptoms (119).

In allergic diseases, the JAK-STAT signaling pathway is influenced by multifaceted and multi-dimensional information, thereby regulating the release of inflammatory factors and the activation of immune cells. The JAK inhibitor CYT387, by inhibiting TSLP-induced dendritic cell maturation, reduces Th2 cell differentiation, significantly lowering the levels of IL-4 and IL-5, which alleviates allergic inflammation. Currently, CYT387 has entered phase II clinical trials, but its long-term safety in AR still needs to be evaluated (120). Precisely regulating the JAK-STAT pathway can effectively suppress Th2-type inflammation and restore barrier function, providing an important strategy for the treatment of allergic diseases.

For allergic reactive lesions, a stratified targeted strategy should be implemented according to the disease staging (see **Table 3**). The implementation of the stratified strategy must be combined with temporal regulation: during the acute inflammatory phase (0-72 hours), priority should be given to inhibiting JAK-STAT (e.g., CYT387 nasal spray) to control Th2 inflammation; in the barrier repair phase (72 hours-2 weeks), Wnt/β-catenin activation should be promoted (e.g., WNT2b-pH sensitive microspheres) to facilitate epithelial regeneration. The time window is based on animal model data: Wnt activation is most effective 48 hours post-injury (113), while JAK inhibitors must be administered within 24 hours after allergen exposure to block STAT6 phosphorylation (120). A dual-drug thermosensitive hydrogel (patent WO2023) can achieve temporal release, and its sustained release kinetics ensure that STAT3 inhibition precedes Wnt activation.

**Table 3:** Targeted treatment strategies for allergic diseases.

I. Acute inflammation phase: control of immune storm					
Target organ	Core pathway	Intervention strategy	Mechanism of action	Representative preparation	Clinical translation stage
Respiratory Tract	JAK-STAT	Inhibition of Th2 Cytokine Signaling	Blocking IL-4/IL-13 induced STAT6 phosphorylation → Reducing eosinophil infiltration	CYT387 Nasal Spray	Phase II Trial (NCT040)
	MAPK	Blocking Epithelial Stress Response	Inhibition of p38 MAPK → Decreasing IL-33 Release	SB203580 Nebulized Solution	Animal Model Validation
Intestinal Tract	RhoA/ROCK	Stabilizing Epithelial Barrier	Inhibition of MLC Phosphorylation → Preventing Tight Junction Disruption	Danshen Ketone Colon-Targeted Microspheres	Phase II for Ulcerative Colitis

II. Barrier repair phase: organizational structure reconstruction					
Respiratory Tract	RhoA/ROCK	Promote Mucus Layer Regeneration	Activate MARCKS Protein → Increase MUC5AC	Secretion Fasudil Sustained-Release Inhalation Powder	Asthma Phase III
Intestinal Tract	Wnt/β-catenin	Accelerate Epithelial Regeneration	Activate LRP5/6 Receptors → Promote β-catenin Nuclear Translocation → Stem Cell Differentiation	WNT2b-pH Sensitive Microspheres	Organoid Model Validation
	TGF-β/Smad	Inhibit Fibrosis	Upregulate Smad7 → Block TGF-βRI Phosphorylation	Pirfenidone Nanoliposomes	Crohn’s Disease Phase II

III. Chronic remodeling phase: maintenance of long-term steady state.					
Systemic	JAK-STAT + Wnt	Temporal Regulation Therapy	First inhibit STAT3 to control inflammation → then activate Wnt to promote repair	Dual-drug thermosensitive hydrogel	Patent Technology (WO2023)
Respiratory Tract	ROCK Inhibitor + Microbiota Regulation	Combined Intervention	Fasudil alleviates airway remodeling + probiotics enhance short-chain fatty acids → synergistically maintain barrier	Co-encapsulated microcapsules	Preclinical synergistic effect

In summary, the five signaling pathways play a crucial regulatory role not only in the pathophysiological processes of mucosal barrier dysfunction but also provide new therapeutic targets for diseases associated with these barrier impairments. Regulatory strategies targeting these signaling pathways should not only inhibit their pathological activation but also consider their physiological roles in barrier repair. This involves not only controlling inflammatory responses but also effectively restoring the stability of the barrier structure. Balancing the activity of these pathways is key to effective treatment. However, previous literature has revealed a scarcity of therapeutic strategies aimed at the synergistic treatment of diseases related to multiple signaling pathways. Future research will delve deeper into the specific mechanisms by which these pathways operate in mucosal barrier diseases. Investigating multi-faceted synergistic regulation is expected to further enhance therapeutic efficacy, achieve comprehensive intervention for barrier damage, and provide a scientific basis for the development of more effective treatment strategies.

Targeting these pathways to treat allergic diseases presents challenges. Common challenges associated with targeting mucosal barrier pathways include pathway redundancy (such as the cooperation between MAPK and JAK-STAT), tissue-specific delivery obstacles, and long-term safety concerns. Future explorations may include local delivery mediated by nanocarriers, temporal regulation (for example, inhibiting JAK-STAT during the acute phase and activating Wnt during the repair phase), or dual-target inhibitors strategies, such as targeting RhoA/ROCK and downstream convergence effectors of MAPK (like MLCK or ERK). Notably, MLCK is regulated by RhoA and is involved in MAPK-mediated cell migration, among other strategies.

## 6 Discussion

The core innovation of this study lies in: ① elucidating the complex mechanisms and potential value of the Wnt/β-catenin, TGF-β/Smad, RhoA/ROCK, MAPK/ERK, and JAK/STAT signaling pathways in maintaining and regulating mucosal barrier function, providing a new perspective for understanding the mucosal barrier that requires a holistic focus to systematically reveal the multi-layered regulatory mechanisms of mucosal barrier function and its dynamic changes under different pathological conditions. The essence of this systemic perspective is to deeply understand the synergistic and antagonistic interactions among the five key signaling pathways emphasized in this paper. ② proposing a ‘staging-organ-targeting’ strategy (**Table 3**), aligning acute immune suppression with regenerative activation during the repair phase.

First, these five signaling pathways demonstrate their synergistic effects in maintaining the homeostasis of the mucosal barrier. The Wnt/β-catenin and TGF-β/Smad signaling pathways directly participate in the formation and repair of the mucosal barrier by regulating the proliferation and differentiation of epithelial cells. The RhoA/ROCK signaling pathway further enhances the physical defense function of the mucosal barrier by regulating the cytoskeleton and TJ proteins. The MAPK/ERK and JAK/STAT signaling pathways play crucial roles in responding to inflammatory responses and immune regulation. The homeostasis of the mucosal barrier requires the normal function of individual signaling pathways, but more importantly, it relies on the dynamic balance and mutual regulation among multiple pathways. This synergistic effect is particularly important in responding to external stimuli, pathogen invasion, and tissue damage.

The results of this study suggest that the signals related to the mucosal barrier are not several independent “linear pathways,” but rather a highly coupled network composed of multiple signaling pathways. In this network, Wnt/β-catenin and TGF-β/Smad jointly regulate epithelial repair and EMT. When Wnt signaling is insufficient, TGF-β/Smad and inflammation-induced MAPK and JAK/STAT activation can still maintain a certain level of epithelial proliferation and matrix remodeling. Conversely, when TGF-β is inhibited, the activation of Wnt/β-catenin and downstream EMT programs can partially compensate for the gap in repair signals. RhoA/ROCK and MAPK directly influence permeability by regulating the actin cytoskeleton and TJ complexes. When RhoA/ROCK is impaired, MAPK-mediated TJ protein phosphorylation and rearrangement can still stabilize the barrier structure to some extent. Furthermore, JAK/STAT can upregulate mucosal inflammatory factors and participate in barrier reconstruction by influencing Wnt/β-catenin and TJ protein expression, providing a certain “buffer zone” between inflammation and repair within the network.The Notch/Hes–Hey, Hippo–YAP/TAZ, and AMPK–mTOR modules further provide upstream integration and compensatory regulation for this network (see **Figure 6**). When Wnt/β-catenin activity decreases, Notch/Hes–Hey can integrate residual Wnt and TGF-β signals to reallocate the fate of intestinal epithelial stem cells, maintaining the ratio of absorptive cells to goblet cells, thereby alleviating the damage to the mucus layer and epithelial integrity. When the RhoA/ROCK or MAPK pathways are impaired, leading to weakened mechanotransduction, Hippo–YAP/TAZ can still sense changes in cell density and local tension, driving stem cell expansion and epithelial regeneration. It compensates for some regulatory functions of the cytoskeleton and TJs through interactions with scaffold proteins related to TJs. In cases where inflammation or abnormal nutritional signals lead to an imbalance in MAPK/JAK/STAT, the AMPK–mTOR axis maintains the turnover of TJ proteins, mitochondrial homeostasis, and mucus/antimicrobial peptide secretion by regulating metabolic status and autophagy levels, thereby providing “compensatory protection” for epithelial survival and barrier function.Through reflection on the above content, it is evident that, from a network perspective, when the function of a certain core pathway is compromised, the remaining core pathways can compensate for its loss through compensatory activation at different levels and targets, thereby maintaining the overall stability of the mucosal barrier to a considerable extent. This perspective also suggests a clinical intervention approach: rather than merely blocking or activating a single pathway, a more valuable strategy may be to reshape the balance of the entire signaling network, allowing compensatory mechanisms to be fully activated while avoiding the long-term overactivation of any single pathway.

On the other hand, it is crucial to be aware of the risks associated with long-term pathway-targeted therapies: JAK inhibitors may increase the likelihood of respiratory infections (infection rate in patients with atopic dermatitis: 10.2%), while the vasodilatory effect of the ROCK inhibitor Fasudil requires monitoring of blood pressure fluctuations (alternative approaches such as local delivery via nasal spray can reduce systemic toxicity by 50%). In contrast, targeted multi-pathway combination therapy demonstrates significant superiority. This approach can simultaneously intervene in multiple key signaling pathways, breaking the resistance mechanisms that may arise from long-term targeting of a single pathway, enhancing the stability and durability of treatment effects, improving patient prognosis, and reducing the risk of progression of allergic diseases.

Therefore, cross-pathway interactions are a core feature of maintaining barrier homeostasis. Cross-pathway interactions are realized through core molecular complexes: for instance, the crosstalk between Wnt and TGF-β indirectly enhances Wnt/β-catenin signaling by inhibiting TGF-βRI phosphorylation via Smad7, while the β-catenin/Smad4 complex synergistically activates key EMT genes (such as Snail), finely regulating the epithelial-mesenchymal transition process. In complex pathological states, the synergy between signaling pathways becomes more pronounced, warranting a focus on the study of the entire signaling pathway network to understand the intricate interactions among these pathways.

The therapeutic strategies formulated for this core feature still face challenges. Intestinal barrier repair primarily relies on Wnt/β-catenin-mediated proliferation and regeneration of epithelial stem cells. In asthma, the upstream IL-4/IL-13-JAK-STAT6 axis, along with TGF-β/Smad signaling, predominantly drives Th2 inflammation and airway remodeling. The RhoA/ROCK-mediated mucus secretion and MAPK-dependent epithelial stress response are more downstream effects of this cytokine network. This is supported by the previous discussion of the underlying mechanisms. The differences arise from tissue-specific microenvironments. Future treatments for allergic diseases need to explore multi-pathway synergistic targeting strategies. For example, regulatory sequences could first inhibit the overactive JAK-STAT pathway to alleviate acute inflammation, followed by the activation of Wnt/β-catenin to promote barrier repair; targeting common nodes shared by different signaling pathways could involve a combined approach targeting RhoA/ROCK and MAPK downstream shared effectors (such as MLCK). It is essential to analyze the differences in the weight of tissue-specific pathways in the future. The existence of this tissue-specific regulatory weight has been demonstrated, but the specific mechanistic differences still require more in-depth research.

This study delves into the role of the aforementioned signaling pathways in the mucosal barrier; however, several limitations remain. First, this research primarily relies on literature focused on *in vitro* experiments and animal models, necessitating further validation of the applicability of these mechanisms in humans. For instance, there are significant differences between the immune cell composition of human intestinal mucosa (such as the proportion of regulatory T cells in the lamina propria), microbial diversity, and the expression profiles of specific receptors (such as TLR4) compared to rodents, which may affect the translational efficacy of pathway regulation. Moreover, there may be differences in the expression levels, activity regulation, or downstream effectors of key signaling pathway molecules (such as certain receptors and kinases) between human cells/tissues and commonly used animal models, which directly impacts the translational potential of preclinical data. Additionally, current research on mucosal barriers has primarily focused on gastrointestinal mucosa, while studies on respiratory mucosa are relatively scarce and require further investigation. Research on respiratory mucosa must pay special attention to its uniqueness: ciliary movement, mucosal viscoelasticity, and the local immune environment (such as the high expression of IL-33) may weaken the barrier repair function of the RhoA/ROCK pathway in asthma compared to the gut. Furthermore, from a clinical practice perspective, targeted therapy for the mucosal barrier still faces three major challenges: (1) the local delivery efficiency of pathway inhibitors (e.g., retention time of nasal drugs); (2) off-target effects caused by tissue-specific expression (e.g., the dual role of RhoA in the airway and vasculature); (3) significant individual differences in microbiota-host interactions that substantially affect pathway activity, such as short-chain fatty acid metabolites regulating Wnt signaling, which leads to variations in barrier repair efficiency among patients. It is suggested to develop predictive models based on microbiota components to match targeted pathway modulators. More critically, the interactions between signaling pathways have not yet been fully elucidated. Future research will focus more on the holistic understanding of mucosal barrier studies, exploring their functional performance and dynamic regulatory mechanisms in different environments. With the development of novel biotechnologies, more potential regulatory mechanisms may be discovered, providing additional options for the treatment of mucosal barrier-related diseases.
